# Successful treatment with azacitidine for the simultaneous occurrence of multiple myeloma and acute myeloid leukemia with concomitant del(5q) and the *JAK2* V617F mutation

**DOI:** 10.1007/s00277-017-3032-8

**Published:** 2017-06-02

**Authors:** Satoko Oka, Kazuo Ono, Masaharu Nohgawa

**Affiliations:** 1 Division of Hematology, Japanese Red Cross Society Wakayama Medical Center, Wakayama, Japan; 2 Division of Pathology, Japanese Red Cross Society Wakayama Medical Center, Wakayama, Japan

**Fig. 1:**
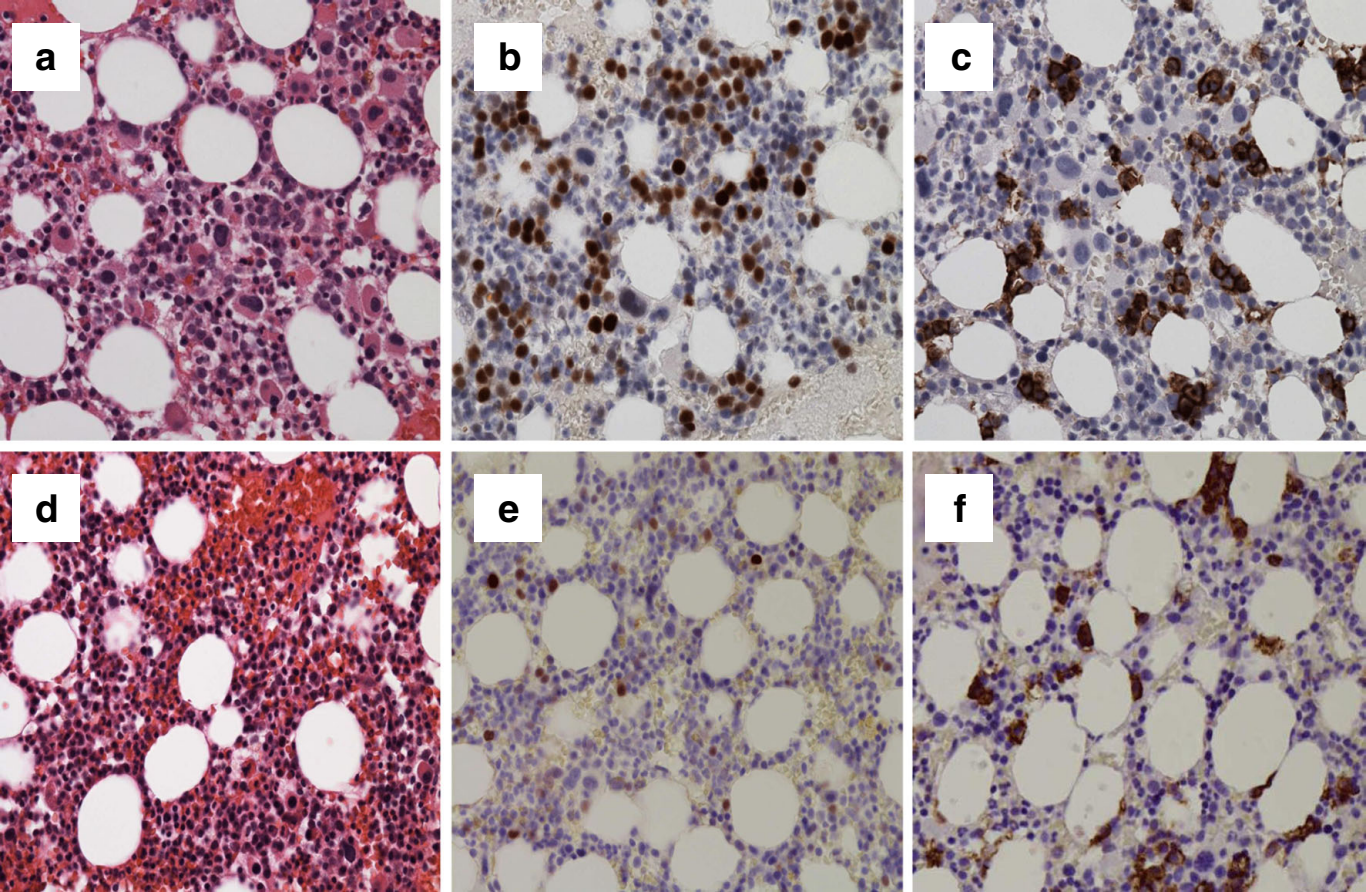
Bone marrow biopsies of the concomitant occurrence of multiple myeloma and acute myeloid leukemia with del(5q) and the *JAK2 V617F* mutation (**a**, **b**, **c**) and after the azacitidine treatment (**d**, **e**, **f**). **a** Hypercellular marrow with myelodysplasia-related changes and numerous blasts. **b** Strong nuclear p53 immunostaining in numerous hematopoietic cells. **c** CD138 immunostaining in 10% of plasma cells. **d** Hypercellular marrow with myelodysplasia-related changes and decreased blasts. **e** Strong nuclear p53 immunostaining in a few hematopoietic cells. **f** CD138 immunostaining in a few plasma cells

Dear Editor,

A 73-year-old female with 5q-syndrome and the co-existent JAK2V617 mutation following 92-month treatment with lenalidomide exhibited evidence of disease progression, with leukopenia (WBC 2.2 × 10^9^/l), anemia (hemoglobin 68 g/l), and 30% blasts in her bone marrow (Fig. 1a). Strong nuclear p53 and CD34 immunostaining was detected in 30 and 10% of hematopoietic cells, respectively (Fig. 1b), and CD138 immunostaining was noted in 10% of plasma cells (Fig. 1c). A FISH analysis revealed 5q31 in 86% of nuclei and molecular analyses were positive for the *JAK2 V617F* mutation (60%). Her serum interleukin (IL)-6 level was 37 pg/ml (normal range, lower than 4 pg/ml). The medullary plasma cell count was <10%. Serum IgG paraprotein and β2-microglobulin (β2-MG) levels increased to 65 g/l (normal range, 7–16 g/l) and 7.8 mg/l (normal range <2 mg/l), respectively. The serum-free light chain ratio was 6.5 (normal range, 0.26–1.25). However, she had no evidence of myeloma-defining events or amyloidosis. A diagnosis of the simultaneous occurrence of acute myeloid leukemia (AML) and smoldering multiple myeloma (MM) was made, and 5-azacytidine (75 mg/m^2^, days 1–7 in a 28-day cycle) was initiated. After 2 cycles of this treatment, she had normal peripheral blood counts and no evidence of circulating blasts. Serum IgG paraprotein, β2-microglobulin (β2-MG) and IL-6 levels decreased to 18 g/l, 2.6 mg/l, and 2.3 pg/ml, respectively. A repeat marrow examination showed slightly hypercellular marrow, with a significant decrease in myeloblasts to 1% blasts and 5% of plasma cells (Fig. 1d). Strong nuclear p53 and CD34 immunostaining decreased in hematopoietic cells (Fig. 1e), and CD138 immunostaining was detected in a few plasma cells (Fig. 1f). Karyotypic analyses showed 46, XX, del(5q)(q13q31) (11/20 cells), 46, XX, del(5q) (q13q31), t(17;21)(q25;q11.2)(9/20 cells). A FISH analysis revealed 5q31 in 20% of nuclei and molecular analyses were positive for the *JAK2 V617F* mutation (8%). The azacitidine treatment has been continued, and the patient maintained hematological CR without the progression of MM in the subsequent 12 months.

Concomitant occurrence of myelodysplastic syndrome (MDS)/myeloproliferative neoplasms (MPN), and MM is a rare event, and the reason of this association remains unclear. The management of these patients involves the treatment of MDS/MPN and the monitoring of MM for transformation to an overt plasma-cell malignancy. However, in patients who develop MM, management is focused on treating myeloma. Agents such as melphalan, thalidomide, lenalidomide, pomalidomide, and bortezomib exhibit clear activity in such patients and need to be considered in the treatment strategy. Azacitidine functions though the proteosomic destruction of DNA methyltransferase and resultant chromatin decondensation, and is not only active in higher-risk disease; similar response rates have been reported in IPSS low/int-1 patients including those with a del(5q) abnormality.

The frequencies of TP53 mutations in MDS and MM were 7–19 and 8–15%, respectively, and TP53 mutant clones may drive disease progression [1–4]. In MDS, p53 nuclear expression has been correlated with hemizygous TP53 mutations, and strong p53 immunostaining in >1% of bone marrow progenitor cells has also been correlated with a higher risk of AML and resistance to lenalidomide therapy [1, 2, 5]. In MM, the presence of TP53 mutations indicates a dismal prognosis, similar to MDS; patients exhibit a more aggressive disease course, more frequently have extramedullary disease and hypercalcemia, and have shorter overall and progression-free survival [3, 4]. In our case, the percentage of strongly p53-positive bone marrow cells was 30% at the time of the concomitant occurrence of leukemic transformation and MM; however, this percentage decreased after the treatment with azacitidine.

Another important distinction pertaining to the origin of these 2 clonal diseases is the involvement of the inflammatory cytokine, IL-6. IL-6 is a potent human myeloma-cell growth factor, and its overproduction is known to play a critical role as an anti-apoptosis-inducing agent in MM. IL-6 also promotes megakaryocytopoiesis and has been implicated in the pathogenesis of MPN [6]. The pathogenetic interactions between IL-6 and putative pluripotent stem cells may also be involved in the pathogenesis of co-existent cases of MM and MPN. Khong et al. recently demonstrated that azacitidine exerts pleiotropic effects including the downregulation of anti-apoptotic factors (IL-6, IL-6 Receptor α, and Bcl-_XL_) and JAK-STAT signaling as well as the inhibition of NFκB in MM cell lines [7]. Serum IL-6 levels increased in our case when SMM developed with the second relapse of AML, and decreased after the treatment with azacitidine.

In our case, azacitidine was effective for MDS transformed to AML and MM after the failure of a long-term treatment with lenalidomide. Although the pathogenetic interactions between IL-6, TP53 mutant clones, and putative pluripotent stem cells are not currently known, IL-6 and TP53 mutations may contribute to the relationship between MDS and MM. Further studies are warranted in order to improve clinical management and biological knowledge.
